# Axial and mean diffusivity predict myelin density in the hippocampus of pigs during early brain development, independent of sex

**DOI:** 10.3389/fnins.2025.1576274

**Published:** 2025-05-19

**Authors:** Loretta T. Sutkus, Zimu Li, Kaitlyn M. Sommer, Ryan N. Dilger

**Affiliations:** ^1^Neuroscience Program, University of Illinois, Urbana, IL, United States; ^2^Department of Animal Sciences, University of Illinois, Urbana, IL, United States

**Keywords:** axial diffusivity, domestic pigs, early-life brain development, hippocampus, mean diffusivity, myelin density, neuroimaging, sex-independent development

## Abstract

**Introduction:**

In the developmental field, sex differences can alter brain growth and development. Across the literature, sex differences have been reported in overall brain volume, white matter, gray matter and numerous other regions and tracts captured through non-invasive neuroimaging. Growing evidence suggests that sex differences appear at birth and continue through childhood. However, limited work has been completed in translational animal models, such as the domestic pig. Additionally, when using neuroimaging, uncertainties remain about which method best depicts microstructural changes, such as myelination.

**Materials and methods:**

To address this gap, the present study utilized a total of 24 pigs (11 intact males or boars; 13 females or gilts) that underwent neuroimaging at postnatal day (PND) 29 or 30 to assess overall brain structural anatomy (MPRAGE), microstructural differences using diffusion (DTI), and an estimation of myelin content via myelin water fraction (MWF). On PND 32, brains were collected from all pigs, with the left hippocampus isolated, sectioned, and stained using the Gallyas silver impregnation method to quantify myelin density.

**Results:**

Minimal sex differences were observed across neuroimaging modalities, with only myelin content exhibiting sex differences in the hippocampus (*P* = 0.022). In the left hippocampus (*P* = 0.038), females had a higher MWF value compared with males. This was supported by histologically derived myelin density as assessed by positive pixel percentage, but differences were isolated to one anatomical plane of the hippocampus (*P* = 0.024) and not the combined mean value (*P* = 0.333). Further regression analysis determined that axial (*P* = 0.01) and mean (*P* = 0.048) diffusivity measures, but not fractional anisotropy or MWF, were positively correlated with histologically derived myelin density in the left hippocampus, independent of sex.

**Discussion:**

These findings suggest that at 4 weeks of age, axial and mean diffusivity may better reflect myelin density. Further investigation is required to confirm underlying mechanisms. Overall, minimal sex differences were observed in 4-week-old domestic pigs, indicating similar brain structure at this early stage of development.

## 1 Introduction

Sex differences across mammals have been a longstanding interest in the field of neuroscience, with much focus placed on adolescence into adulthood (Perrin et al., 2009; Lenroot and Giedd, 2010; Gur and Gur, 2016). Divergence of brain development between males and females, such as larger brain volumes in males and differences in white matter development, have been primarily tied to hormonal fluctuations during puberty in humans (Lenroot and Giedd, 2010; Gur and Gur, 2016). Although substantial evidence suggests developmental differences emerge as early as *in utero*, driven by variations in cellular concentrations, metabolic rates, and sex chromosomes (Ray et al., 1995; Lenroot and Giedd, 2010), research into human fetal infant brain development remains limited and controversial (Saker et al., 2024). Across infancy and throughout adulthood, multiple sex-dependent variances have been reported in total and regional brain volumes (Dean et al., 2018; Khan et al., 2024), cerebral lobe volumes (Lehtola et al., 2019), and brain growth patterns (Lehtola et al., 2019). However, others have reported little to no sex-specific differences at this early stage of human brain development when utilizing neuroimaging methods such as diffusion tensor imaging (Kumpulainen et al., 2023). In preclinical models, neurological sex differences at birth and throughout childhood have been reported in rats (Levine and Mullins, 1966; Kühnemann et al., 1994), guinea pigs (Resko and Roselli, 1997; Bartesaghi et al., 2003), and non-human primates (Resko and Roselli, 1997; Knickmeyer et al., 2010; Scott et al., 2016). Meanwhile in other species, like ferrets (Garcia et al., 2024) and cats (Villablanca et al., 2000), no sex differences in early brain development were apparent. Nonetheless, previous work has determined a critical period between prenatal and early postnatal development during which exposure to testosterone influences brain development in rats (Levine, 1966). Therefore, further investigation into early-life sex driven neurological differences remains a priority.

An emerging biomedical model that has gained traction in the field of neuroscience is the domestic pig. Especially when utilizing neuroimaging, the pig has been acknowledged as an advantageous preclinical animal model due to their large brain size, gyrencephalic anatomical structure, and comparable cerebral structures (Lind et al., 2007; Sauleau et al., 2009). Previously, sexual dimorphisms have been explored in domestic pigs, revealing differences in brain gene expression (Strawn et al., 2021), neurotransmitter concentrations (Henry et al., 1996), overall brain growth and volumes (Conrad et al., 2012a), and exploratory behaviors (Fleming and Dilger, 2017). Additionally, variation in gene expression and signaling pathways have been observed between male and female pigs as early as the 6th week of gestation (Strawn et al., 2021). The 4-week postnatal time period, in particular, has been determined to be critical for rapid brain development in the pig (Fil et al., 2021a), with behavioral sex differences already apparent at this age (Fleming and Dilger, 2017). Though sex specific differences in cortical volume, hippocampus, and cerebellum were only observed at 24-weeks of age (Conrad et al., 2012a), it is possible that the limited sensitivity of the neuroimaging methods utilized did not fully capture earlier variation. Overall, research into early-life sexual dimorphisms in domestic pigs remains limited and warrants further exploration to better understand the translational power of the pig.

A major limitation in current developmental research using neuroimaging is the uncertain sensitivity of various neuroimaging modalities for assessing white matter properties. Across various modalities, there are ongoing debates and criticism of the accuracy with which each method predicts biological mechanisms, particularly those reflecting myelination (Mädler et al., 2008; Jones et al., 2013; Lee et al., 2021). Commonly used myelin sensitive techniques, such as diffusion and myelin water imaging, have been highly debated because of concerns in their reflection of ground-truth myelin density. To address these challenges, the current study paired neuroimaging data with histological methods to establish ground-truth measurements and assess their alignment with image-derived values. In addition to this validation effort, the primary objective of the study was to investigate sex differences in brain development during early life in the domestic pig. Specifically, multimodal neuroimaging methods were applied to capture macrostructural, microstructural, and myelination variations in the young pig. The hippocampus was selected as a region of interest due to its sensitivity to sex differences (Yagi and Galea, 2019; Van Eijk et al., 2020) and for its crucial role in memory and developing behaviors (Rubin et al., 2014).

## 2 Materials and methods

Approval from the University of Illinois Urbana-Champaign Institutional Animal Care and Use Committee was obtained prior to conducting all experimental procedures described and were in congruence with the Guide for the Care and Use of Laboratory Animals.

### 2.1 Animal care and housing

On postnatal day (PND) 2, 11 intact male (i.e., non-castrated; boars) and 13 female (i.e., gilts) pigs (*N* = 24) were sourced from a commercial swine herd [Pig Improvement Company (PIC; Hendersonville, TN) breeding females (i.e., sows) Line 3 dams artificially inseminated using a pooled semen source from 50 to 150 boars] across two separate cohorts. Throughout the study, pigs were artificially reared in the University of Illinois Piglet Nutrition and Cognition Lab (PNCL) in individual home-cage units that allowed pigs to see, hear, and smell each other, but prevent direct contact. Unit specifications and housing conditions were reported previously (Fil et al., 2021a). Pigs were randomly assigned to an individual home-cage based on litter of origin, body weight, and sex. All pigs received a 5-mL subcutaneous and 3-mL oral dose of *Clostridium perfringens* antitoxin C and D (Colorado Serum Company, Denver, CO) at the beginning of the study and received *ad libitum* access to a nutritionally adequate milk replacer (Multi-Species Milk Replacer, Purina Animal Nutrition LLC, North Arden Hills, MN) until study conclusion on PND 32.

### 2.2 Magnetic resonance imaging (MRI) procedures

On PND 29 or 30, all 24 pigs (11 males/boars; 13 females/gilts) underwent neuroimaging procedures at the Biomedical Imaging Center (Beckman Institute for Advanced Science and Technology, University of Illinois at Urbana-Champaign, Urbana, IL). Using a Siemens MAGNETOM Prisma 3T MRI, three sequences were included in the neuroimaging protocol: (1) a magnetization-prepared rapid gradient-echo (MPRAGE) sequence for assessment of structural volume; (2) a diffusion tensor imaging (DTI) sequence to assess fiber structure organization; and (3) a multicomponent-driven equilibrium single pulse observation of T_1_ and T_2_ (mcDESPOT) sequence to obtain myelin-associated water fraction (MWF) for myelin content quantification.

All pigs were fasted for 3–5 h before neuroimaging to avoid anesthetic complications. Prior to imaging acquisition, pigs were prepped for endotracheal intubation by first sedating them with an intramuscular injection of a telazol:ketamine:xylazine (TKX) cocktail [50.0 mg tiletamine plus 50.0 mg of zolazepam reconstituted with 2.50 mL ketamine (100 g/L) and 2.50 mL xylazine (100 g/L); Fort Dodge Animal Health, Overland Park, KS] at 0.04–0.05 mL/kg of body weight. Immobilization was checked by jaw tone, palpebral reflex, and hind hoof reflex. After anesthetic plane sedation was confirmed, the pig was fitted with earplugs, Puralube vet ointment (Dechra Veterinary Products, Overland Park, KS) was administered to both eyes, eyes were secured with lightweight cloth surgical tape, and temperature was recorded. Using a veterinary laryngoscope (DarvallVet Advanced Anesthesia Specialists, Gladesville, NSW, Australia) and atomizer, 1 mL of lidocaine was sprayed on the arytenoid cartilages to prevent laryngospasms. After 2–3 min, the pig was intubated with a 3 mm cuffed endotracheal tube.

Once proper intubation was confirmed, pigs were placed in a supine position, their head fitted into a 15-channel knee coil (1 transmit/15 receive knee coil for Prisma from QED), and the endotracheal tube was connected to an anesthesia machine (Dream; Vetamac, Inc. Rossville, IN) via a Y-circuit tube connection. Pigs were maintained under surgical anesthesia using isoflurane (98% O_2_/1%−2% isoflurane) throughout the duration of the one-h scan. Heart rate, partial pressure of oxygen (PO_2_), and end tidal CO_2_ were monitored and recorded every five min utilizing two infrared sensor pulse oximeters (LifeWindow LW9x, Boynton Beach, FL and MEDRAD Veris 8600, Indianola, PA) attached to the pig's hind hoof and/or tail. Based on recorded vital signs and capnography readings, manual breaths were delivered via a 1-L rebreathing bag and adjustments to percent isoflurane were made as necessary.

#### 2.2.1 MPRAGE acquisition and analysis

For anatomical assessment of the brain, MPRAGE sequences were acquired in sagittal orientation from the tip of the snout to the cervical/thoracic spinal cord junction. Images were acquired with a 173 mm × 173 mm × 153.6 mm field of view (FOV) and a 288 × 288 × 256 matrix size. Scans were captured with the following specifications: repetition time (TR) = 2,060 ms, echo time (TE) = 2.05 ms, inversion time (TI) = 1,060 ms, and a flip angle (α) = 9°. This provided a final voxel volume of 0.6 × 0.6 × 0.6 mm^3^. Image processing primarily followed procedures described by Golden et al. (2024) other than for the brain extraction and tissue segmentation protocols, resulting in 28 regions of interest (ROI) and seven combined whole ROI identified for each subject.

For brain extraction, we used a 2.5D deep learning-based segmentation method. This method combined adjacent slices into three channels and employed a U-Net architecture with an EfficientNetB5 backbone (Ronneberger et al., 2015; Tan and Le, 2019). Encoders pre-trained on ImageNet were used for feature extraction (Deng et al., 2009). We trained a set of models using a dataset of 116 4-week-old young pig MPRAGE images obtained from previous studies (Fil et al., 2021a,b; Golden et al., 2024; Joung et al., 2020; Sutkus et al., 2022). Inference was performed on sagittal, coronal, and axial views, and results were combined using a majority-voting ensemble. All brain masks underwent a thorough quality control process by trained technicians to ensure accuracy.

Tissue segmentation was conducted using FSL FAST (FMRIB's Automated Segmentation Tool; Zhang et al., 2001). This approach has been previously used in non-human animal models, as shown by Messinger et al. (2021). To improve segmentation accuracy in young pig brains, a tissue probability map specific to young pigs, from Fil et al. (2021b), was used as a prior for FSL FAST. This resulted in segmentations for gray matter (GM), white matter (WM), and cerebrospinal fluid (CSF). From these segmentations, intracranial volume (ICV) and total brain volume (TBV) were calculated.

#### 2.2.2 Diffusion tensor imaging (DTI) acquisition and analysis

A diffusion-weighted echo planar imaging (DWI-EPI) sequence (Feinberg et al., 2010; Moeller et al., 2010; Xu et al., 2013) was acquired for assessment of white matter microstructure. Images were obtained in transverse orientation with a 160 mm × 160 mm × 80 mm field of view (FOV) and a 100 × 100 × 50 matrix size. Scans were obtained using the following parameters: TR = 5,100 ms, TE = 70 ms, α = 90°, GRAPPA accelerated by a factor of 2 in the phase encode direction, multiband factor of 1, and 3 diffusion weightings at 0, 1,000, and 2,000 s/mm^2^ across 30 directions.

Raw DWI images were subjected to denoising and motion correction utilizing workflows within the DIPY toolbox (Garyfallidis et al., 2014). For denoising, the Marchenko-Pastur Principal Component Analysis (MPPCA) workflow was utilized (Manjón et al., 2013; Veraart et al., 2016a,b; Neto Henriques, 2018), whereas for motion correction an affine registration method was used (Jenkinson and Smith, 2001). Afterwards, skull stripping was performed using the same automated method as MPRAGE but with a model trained on 128 images obtained from previous studies (Fil et al., 2021a,b; Golden et al., 2024; Joung et al., 2020; Sutkus et al., 2022). All resulting brain masks were quality-controlled by trained technicians to ensure accuracy.

**Figure 1:**
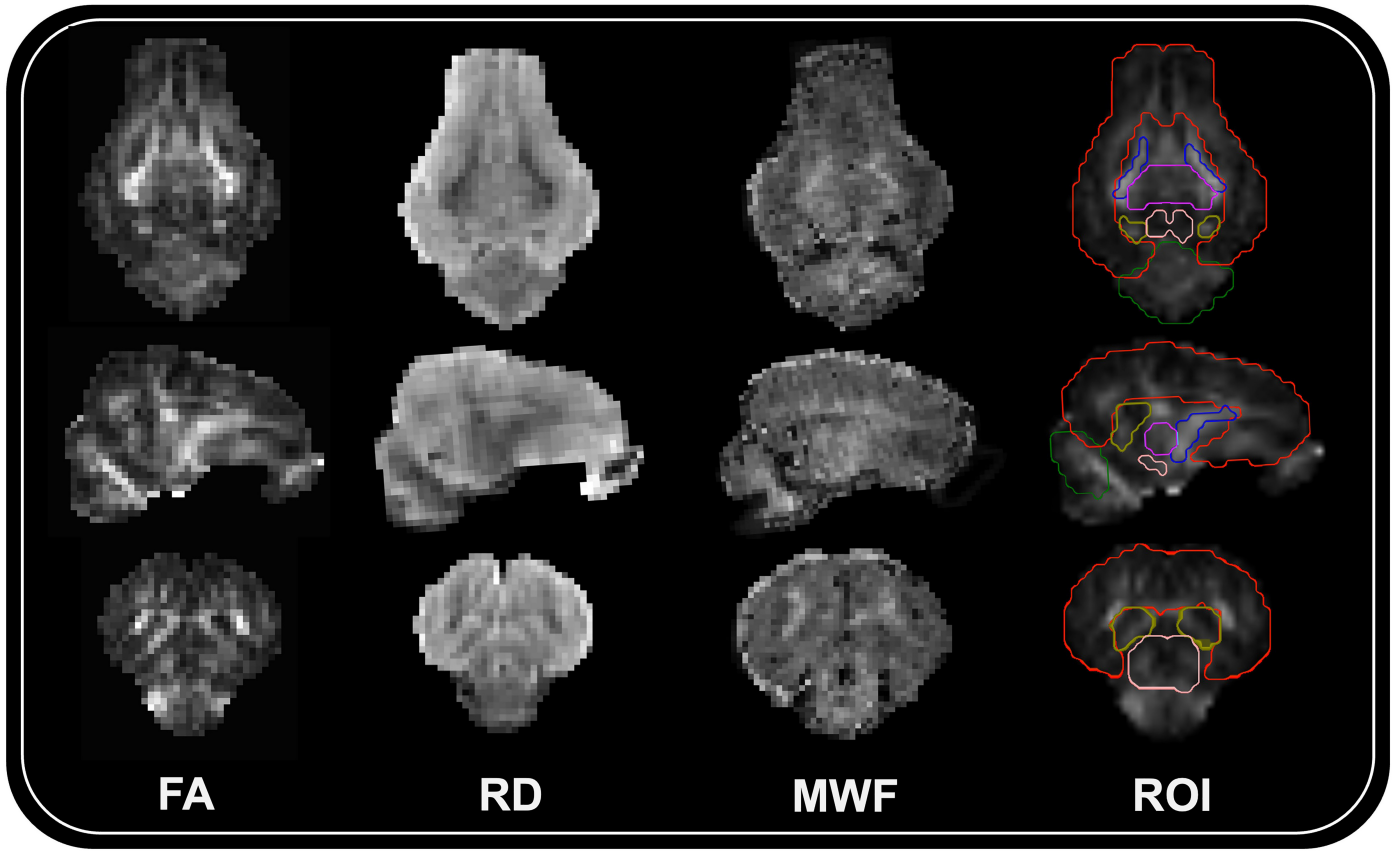
Representative neuroimaging subject maps and ROI registration. Representative maps for fractional anisotropy (FA), radial diffusivity (RD), myelin water fraction (MWF), and region of interest registration (ROI) overlayed on the FA subject map are displayed for the same female pig. From the top to bottom are transverse, sagittal, and coronal sections of the brain. Six representative ROI boundaries (red = cortex; blue = internal capsules; purple = thalamus; peach = midbrain; yellow = hippocampus; green = cerebellum) are displayed on the ROI registration image.

Then, using the diffusion model within DIPY (Basser et al., 1994; Pajevic and Pierpaoli, 1999), diffusion tensors were reconstructed to produce maps of fractional anisotropy (FA), axial diffusivity (AD), radial diffusivity (RD), and mean diffusivity (MD). Using advanced normalization tools (ANTS; Avants et al., 2014), each subject's reconstructed FA map was then registered to their corresponding MPRAGE image. This allowed for ROI within MPRAGE space to easily be warped into diffusion space. After registration, mean FA, AD, RD, and MD values were extracted for the following ROI: corpus callosum, left/right caudate, left/right cortex, left/right hippocampus, left/right internal capsule, left/right putaman and globus pallidus, and the thalamus. Representative maps for FA and RD, as well as ROI registration to a subject brain is displayed in Figure 1.

#### 2.2.3 Myelin water fraction (MWF) acquisition and analysis

To estimate myelin content across the brain, a myelin water imaging sequence called multicomponent-driven equilibrium single pulse observation of T_1_ and T_2_ (mcDESPOT; Deoni et al., 2008; Deoni, 2011; Deoni and Kolind, 2015) was acquired. This technique involves multiple sets of spoiled gradient-recalled echo (SPGR) and T_1_/T_2_-weighted balanced steady-state free precession (SSFP) sequences that were acquired at various flip angles at a constant TR. The sequences were obtained in sagittal orientation with a 160 mm × 160 mm × 124.8 mm FOV and 128 × 128 × 96 matrix size. SSFP sequences involved two phase-cycling increments of 0° and 180° that were acquired with the following parameters: TR/TE = 5.3 ms/2.7 ms, α = (11, 15, 19, 23, 27, 35, 50, and 70)°, and bandwidth = 350 Hz/Px, to achieve a final voxel volume of 1.3 × 1.3 × 1.3 mm^3^. SPGR sequences were acquired with the following parameters: TR/TE = 5.6 ms/2.7 ms, α = (3, 4, 5, 6, 7, 9, 13, and 18)°, and bandwidth = 350 Hz/Px, providing a final voxel volume of 1.3 × 1.3 × 1.3 mm^3^. Two additional high-resolution T_1_-weighted inversion recovery (IR)-SPGR sequences were also acquired for correction of transmit (B_1_) magnetic field inhomogeneities. These sequences were obtained utilizing the following parameters: TR/TE = 5.6 ms/2.7 ms, α = 5°, and bandwidth = 350 Hz/Px, to achieve a final voxel volume of 1.7 × 1.7 × 2.6 mm^3^. Image processing and analysis followed previously reported procedures (Golden et al., 2024). A representative MWF map as derived from mcDESPOT image processing is displayed in Figure 1.

### 2.3 Brain collection and Gallyas silver impregnation staining

At study conclusion, on PND 32, pigs were sedated via an intramuscular injection of the TKX cocktail at 0.06–0.08 mL/kg of body weight. Palpebral and hind hoof reflexes were checked to ensure a surgical level of anesthesia. Once full sedation was confirmed, pigs were euthanized by delivering an intracardiac injection of sodium pentobarbital (Euthasol, Virbac, Wetlake, TX, USA) at 0.22 mL/kg of body weight. The brain was then extracted (Fleming et al., 2021), separated by hemisphere, and both sections were immersion-fixed in 10% neutral-buffered formalin (NBF) which has been previously reported to retain tissue quality sufficiently for staining (Sharma and Grieve, 2006; McFadden et al., 2019). Hemispheric sections remained in 10% NBF for 7 days to preserve tissue morphology, at which point they were transferred to 0.1% sodium azide in phosphate-buffered saline (PBS) for stabilization until sectioning. To prepare for sectioning, the left hemisphere samples were dissected to isolate the hippocampus. Each sample was cut rostral to the corpus callosum, trimmed caudally to expose the caudal-most part of the hippocampus, and trimmed laterally to form a 1–2-cm block of tissue. Next, the sample was successively immersed until sinking in gradients of 10%, 20%, and 30% sucrose in PBS. Upon sinking in the 30% gradient, the tissue block was removed, solidified in dry ice, and sectioned caudally into 60-μm thick sections with a CM1950 cryostat (chamber temperature at −25°C; Leica Biosystems, Deer Lake, IL). Sectioned tissue slices were subsequently transferred to 0.1% sodium azide in PBS to stabilize until staining.

**Figure 2:**
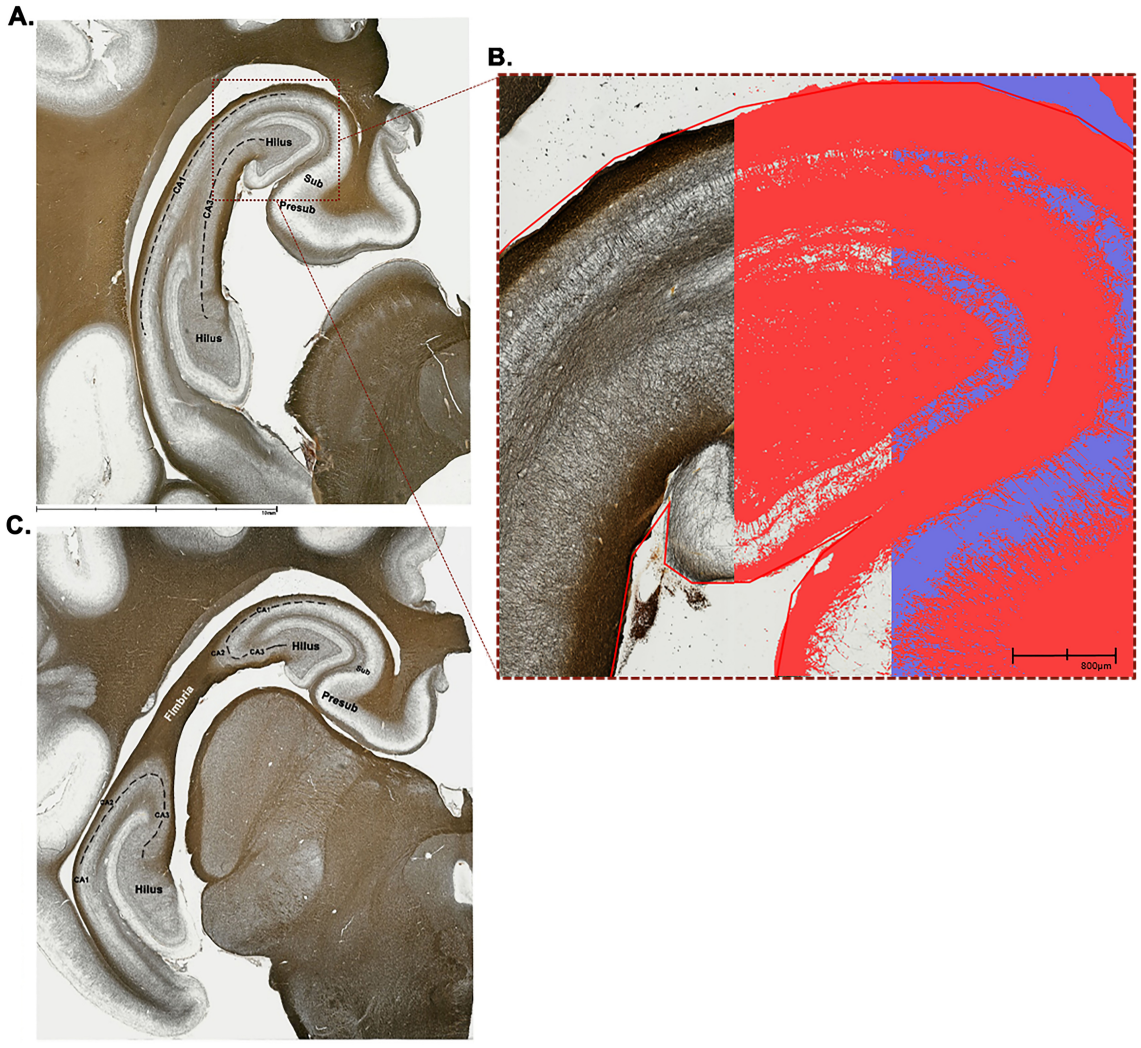
Anatomical representations and application of positive pixel threshold to stained left hippocampal tissue. Two different anatomical positions were selected for staining, a section more caudal **(A)** and another more rostral **(C)**. Both sections included the hilus, CA1, CA3, subiculum, and presubiculum subfields, whereas the more rostral section **(C)** also included CA2 and the fimbria. All anatomical subfield boundaries denoted in this figure are rough estimations that were drawn based on reviewing the following literature (Blackstad et al., 2022; Holm and Geneser, 1991; Holm and West, 1994; Saito et al., 1998; Severi et al., 2005; van der Beek et al., 2004). Tissue sections were 60 μm thick and were stained with the Gallyas silver impregnation method to isolate individual myelin fibers. To quantify myelin fiber density, the positive pixel percentage approach was utilized. A trained technician set a threshold for each subject's image to ensure that the dark stained myelin fibers are captured as “positive” pixels (red color) and the light-colored background as “negative” pixels (purple color). **(B)** Pictured is a close-up view of the position 1 tissue **(A)** where the left-most panel visualizes the dark stained myelin fibers, the central panel showcases when a threshold was applied to capture only positive pixels (red color), and the right-most panel captures both positive (red) and negative (purple) pixels. CA, cornu ammonis; Presub, presubiculum; Sub, subiculum.

Forty-eight h before staining, tissue sections were transferred to 1%−2% paraformaldehyde solution for post-fixation. Four representative tissue sections representing two different anatomical points of the hippocampus were selected for each subject. To ensure that tissue section selection was as consistent as possible between subjects, key anatomical features were identified following Figure 2. These two representative anatomical sections were selected so that major hippocampal subdivisions (i.e., hilus, CA1–CA3, presubiculum, subiculum, and fimbria) could be visually distinguished (Holm and Geneser, 1991; Holm and West, 1994; Saito et al., 1998; van der Beek et al., 2004).

Approximately 16–18 h before staining, selected tissue slices were mounted onto gelatin subbed, frosted tip microscope slides (25 mm × 75 mm; Corning Incorporated, Tewksbury, MA) and left in a 39°C incubator overnight. The following morning, myelin-specific staining was performed following the Gallyas silver impregnation technique procedures previously described (Pistorio et al., 2006; Singh et al., 2024). For the pre-mounted slides, all tissue were stained for 5 min, but timing of clearing varied depending on visual inspection of each tissue sample. In cases where tissues were visually overstained as described by Singh et al. (2024), clearing was repeated until optimal staining was achieved. The next day, stained slides underwent a 5-min xylene wash and were coverslipped with cover glasses (Corning Inc., Corning, NY) using mounting medium (Permount; Thermo Fisher Scientific Inc., Waltham, MA).

### 2.4 Myelin density quantification via positive pixel analysis

Gallyas silver-stained hippocampal slides were scanned using the C9600 Series NanoZoomer-HT (Hamamatsu Photonics, Hamamatsu City, Shizuoka, Japan). The scanner was set to 40 × magnification following established operational guidelines (Carl R. Woese Institute for Genomic Biology, University of Illinois at Urbana-Champaign, Urbana, IL). Slides were processed automatically, and digital images were saved in NDPI format for further analysis.

Scanned NDPI files were loaded into QuPath (version 0.5.1; Bankhead et al., 2017) for image analysis. Regions of interest (ROIs) were manually annotated for the left hippocampus using the polygon tool. Two separate ROIs were drawn for each slide, corresponding to the two different anatomical positions of the left hippocampal tissue as visualized in Figure 2. Regions of avoidance (ROAs) were also manually annotated to exclude areas that were overstained, contained tissue rips/tears, or overlapped with other tissue that are not considered part of the hippocampus. All annotations, including ROIs and ROAs, were organized and saved within the QuPath project hierarchy.

Next, the pixel classification function within QuPath was utilized to create a positive pixel threshold for quantifying myelin density in the hippocampal tissue as visualized in Figure 2. The pixel classifier was set to combine the red, green, and blue channels into a single grayscale image. A Gaussian filter with a smoothing sigma of 1.5 was applied to reduce noise and enhance the visibility of dark-stained regions. The threshold was adjusted iteratively by a trained technician who visually inspected the classifications to ensure that dark-stained regions were correctly identified as positive and the light background as negative. For each image, technicians evaluated and adjusted the threshold as necessary to ensure accurate classification across all ROIs. Positive pixel percentages were subsequently exported for statistical analysis.

### 2.5 Statistical analysis

To determine sufficient treatment sample size, a power analysis was conducted with 80% power and a 5% level of significance (i.e., alpha = 0.05) using SAS (RRID:SCR_008567; version 9.3; SAS Inst. Inc., Cary, NC, United States) prior to study initiation. Statistical analyses were performed to evaluate differences in neuroimaging and histological outcomes by sex and to determine which MRI-derived metrics correlate with histology derived myelin density in the left hippocampus. Neuroimaging and positive pixel percentage outcomes were analyzed using SAS (RRID:SCR_008567; version 9.3; SAS Inst. Inc., Cary, NC, United States) and Python (version 3.11). In Python, the following libraries and packages were utilized: pandas for data manipulation, numpy for numerical operations, seaborn and matplotlib for visualization, and statsmodels for mixed-effect models and regression analysis (Hunter, 2007; McKinney, 2010; Seabold and Perktold, 2010; Harris et al., 2020; Waskom, 2021).

#### 2.5.1 Groups comparisons

The main effect of sex for each outcome was assessed via a one-way analysis of variance (ANOVA) with the MIXED procedure in SAS. To control for variation due to genetic factors (i.e., dam/litter of origin) and environment (i.e., contemporary group of pigs), cohort was included as a random effect with sow identifier nested within cohort. Outliers were identified and excluded when studentized residuals were found to exceed an absolute value of 3 and overall significance was accepted at *P* < 0.05. Violin plots were utilized for group comparisons to visualize numerical distributions of data, as well as median values and interquartile ranges. Within these violin plots the width of the curves correspond to the density of data points at each value, where wider sections correspond to higher density.

#### 2.5.2 Regression analysis

Generalized linear regression models were employed to explore the relationship between neuroimaging metrics and histological myelin density in the left hippocampus. Separate models were created for DTI-derived metrics and MWF outcomes. Each regression model included sex as a categorical predictor (encoded as a classification variable) and interaction terms of MRI metric × sex to examine sex-specific effects. Cohort and dam identifier were included as random effects in mixed-effects regression models to account for environmental and genetic variability, respectively. Model assumptions of normality, homoscedasticity, and independence were evaluated using residual diagnostics. After fitting the models using the restricted maximum likelihood (REML) method, model comparisons were performed using Akaike's information criterion (AIC) and Bayesian information criterion (BIC) to identify the best-fitting predictor (Maestrini et al., 2024). Additionally, marginal and conditional *R*^2^ were calculated for each model as estimators of explained variance from both fixed and random effects (Nakagawa and Schielzeth, 2013).

## 3 Results

### 3.1 General outcomes

Across the various neuroimaging procedures, data from several subjects (*n* = 1 for DTI; *n* = 3 for MWF) were excluded due to acquisition issues, such as excessive noise or improper field of view caused by animal movement. The final sample sizes for each neuroimaging sequence are reflected in the corresponding tables and figures.

#### 3.1.1 Volumetric outcomes

**Figure 3:**
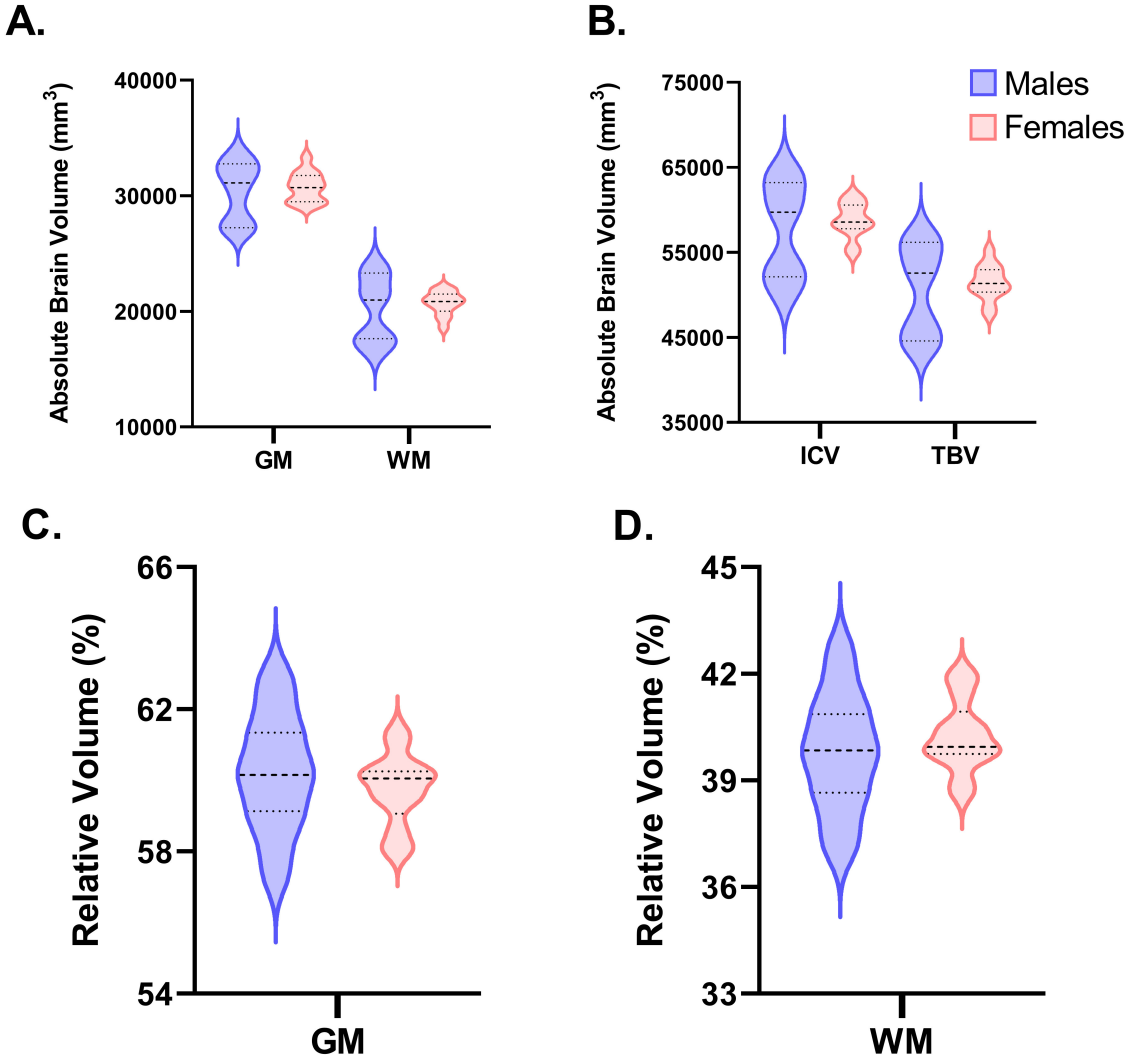
Volumetric comparisons for GM, WM, ICV, and TBV between male (*n* = 11) and female (*n* = 13) pigs. The following violin plots highlight the distributions between male and female pigs in absolute and relative volumes in select brain regions. Wider curves correspond to a higher density of data points at that specific value. The bold dashed line reflects the median value, whereas the other two dashed lines above (3rd quartile) and below (1st quartile) the median reflect the interquartile range. **(A, B)** Display the variance between males and females for absolute volume in gray matter (GM), white matter (WM), intracranial volume (ICV) and total brain volume (TBV). No differences were observed between sexes (*P* < 0.05), although the variance between individual pigs among male pigs seems to be more widespread than female pigs. **(C, D)** Represent the relative volume of GM and WM to TBV. Again, no sex differences were observed, and variation was greater for male vs. female pigs.

Volumetric data for ICV, TBV, GM, WM, CSF, 28 individual ROI across the right and left hemisphere, and seven combined whole ROI are shown in Figure 3. ICV reflects the combination of GM, WM, and CSF, whereas TBV is the combination of GM and WM. Across both ICV (*P* = 0.887) and TBV (*P* = 0.978), there were no differences driven by sex with average absolute volumes being nearly identical for females (ICV = 58,612 mm^3^; TBV = 51,476 mm^3^) and males (ICV = 58,951 mm^3^; TBV = 51,538 mm^3^). Similarly, across GM (*P* = 0.993), WM (*P* = 0.918), and CSF (*P* = 0.354) no sex-driven differences in absolute volume were observed. Likewise, across all 28 individual ROI and when combined to reflect the whole ROI, no differences (*P* > 0.05) were found in absolute volume between males and females. When corrected for ICV, similar results were observed where females and males exhibited comparable ROI volumes relative to ICV across GM, WM, CSF, and almost all selected ROI. A difference was found for the superior colliculus where female pigs were observed to have a higher (*P* = 0.048) relative volume (0.77%) compared with male pigs (0.74%). This difference may be an indication of asymmetry within the brain, as it was mostly localized to the relative volume of the superior colliculus in the right hemisphere (males = 0.37%; females = 0.39%; *P* = 0.051). In the left hemisphere, relative superior colliculus volume was nearly the same across sexes (males = 0.37%; females = 0.38%; *P* = 0.154). GM and WM volume were also corrected for TBV, in which no significant differences were found between sexes sharing the same level of significance (*P* = 0.468). In general, both sexes exhibited a similar GM-to-WM-ratio of ~60:40 (GM: males = 60.17%, females = 59.73%, SEM = 0.427; WM: males = 39.83%, females = 40.27%, SEM = 0.427). All absolute and relative volume outcomes are displayed in Supplemental Table 1 and Table 1, respectively.

**Table 1:** Sex differences across relative volume in 4-week pigs^1^.

**ROI**	**Sex**		**Pooled SEM**	***P*-value**
	**Males/boars**	**Females/gilts**		
*n*	11	13	–	–
Gray matter (ICV)	52.78	52.27	0.369	0.333
White matter (ICV)	35.03	35.26	0.478	0.732
Gray matter (TBV)	60.17	59.73	0.427	0.468
White matter (TBV)	39.83	40.27	0.427	0.468
Cerebral spinal fluid	12.34	12.52	0.271	0.638
Combined caudate	1.32	1.30	0.017	0.467
Combined cortex	63.05	62.96	0.305	0.826
Combined hippocampus	1.76	1.73	0.030	0.382
Combined inferior colliculi	0.44	0.43	0.009	0.563
Combined internal capsules	2.18	2.22	0.019	0.184
Combined putamen and GP	0.69	0.70	0.010	0.448
Combined superior colliculi	0.74^a^	0.77^b^	0.010	0.048
Cerebellum	12.00	12.17	0.152	0.426
Cerebral aqueduct	0.08	0.09	0.002	0.219
Corpus callosum	0.85	0.81	0.020	0.171
Fourth ventricle	0.19	0.19	0.008	0.894
Hypothalamus	0.23	0.24	0.009	0.313
Lateral ventricle	1.08	1.04	0.028	0.265
Left caudate	0.68	0.67	0.011	0.450
Left cortex	32.04	32.09	0.147	0.809
Left hippocampus	0.89	0.87	0.017	0.312
Left inferior colliculus	0.22	0.21	0.006	0.701
Left internal capsule	1.17	1.18	0.015	0.608
Left olfactory bulb	1.93	1.89	0.081	0.582
Left putamen-globus pallidus	0.35	0.36	0.007	0.458
Left superior colliculus	0.37	0.38	0.005	0.154
Medulla	3.32	3.30	0.048	0.627
Midbrain	4.05	4.05	0.035	0.941
Nucleus accumbens	0.09	0.09	0.003	0.998
Pons	2.70	2.74	0.040	0.546
Right caudate	0.64	0.63	0.008	0.616
Right cortex	31.41	31.26	0.210	0.508
Right hippocampus	0.87	0.86	0.014	0.652
Right inferior colliculus	0.22	0.22	0.005	0.539
Right internal capsule	1.01	1.04	0.010	0.073
Right olfactory bulb	1.96	1.94	0.058	0.705
Right putamen-globus pallidus	0.34	0.34	0.006	0.643
Right superior colliculus	0.37	0.39	0.006	0.051
Substantia nigra	0.07	0.07	0.002	0.520
Thalamus	2.66	2.65	0.028	0.888

^1^Data presented are least squares means and *P*-values from mixed model 1-way ANOVA.

^a, b^Means lacking a common superscript letter within a row differ (*P* < 0.05).

ROI, region of interest; SEM, standard error of the mean; GP, globus pallidus; ICV, intracranial volume; TBV, total brain volume.

#### 3.1.2 Diffusion tensor imaging outcomes

The metrics derived from DTI correspond to the diffusion of water molecules moving parallel (i.e., AD) to fibers, perpendicular (i.e., RD) to fibers, overall on average (i.e., MD), and in a specific orientation (i.e., FA). Across all diffusion outcomes, no significant differences (*P* > 0.05) were observed between females/gilts and males/boars (Supplemental Tables 2–4). In general, all values were consistent across regions in both sexes, hence this description will focus on general trends across the diffusion metrics.

For FA (Table 2), white matter regions like the internal capsule were observed to have the highest values (males = 0.441; females = 0.437; *P* = 0.761), with the corpus callosum, thalamus, and putamen exhibiting similar values (between 0.2 and 0.3). Mixed WM and GM regions like the hippocampus and overall cortex exhibited lower values (between 0.1 and 0.2). On the other hand, when investigating RD, the corpus callosum was observed to have the highest average values (males = 0.654 mm^2^/s; females = 0.650 mm^2^/s; *P* = 0.897), whereas internal capsules had the lowest values (males = 0.403 mm^2^/s; females = 0.405 mm^2^/s; *P* = 0.850). A similar trend was observed for MD where the corpus callosum (males = 0.742 mm^2^/s; females = 0.738 mm^2^/s; *P* = 0.911) had the highest reported values and internal capsule (males = 0.550 mm^2^/s; females = 0.552 mm^2^/s; *P* = 0.873) had the lowest. Meanwhile for AD, the corpus callosum still retained the highest values (males = 0.918 mm^2^/s; females = 0.914 mm^2^/s; *P* = 0.925), while the thalamus was observed to have the lowest values (males = 0.756 mm^2^/s; females = 0.745 mm^2^/s; *P* = 0.545). These trends may serve as a baseline for expected diffusion outcomes at this stage of development in young pigs.

**Table 2:** Sex differences in fractional anisotropy of 4-week pigs^1^.

**ROI**	**Sex**		**Pooled SEM**	***P*-value**
	**Males/boars**	**Females/gilts**		
*n*	11	12	–	–
Corpus callosum	0.233	0.236	0.012	0.759
Hippocampus	0.177	0.174	0.004	0.616
Internal capsules	0.441	0.437	0.011	0.761
Left caudate	0.182	0.182	0.008	0.960
Left cortex	0.130	0.129	0.003	0.693
Left hippocampus	0.173	0.177	0.008	0.628
Left internal capsule	0.434	0.437	0.012	0.856
Left putamen and GP	0.253	0.269	0.012	0.368
Putamen and globus pallidus	0.275	0.274	0.013	0.926
Right caudate	0.177	0.180	0.007	0.717
Right cortex	0.134	0.131	0.002	0.232
Right hippocampus	0.171	0.171	0.007	0.948
Right internal capsule	0.437	0.436	0.018	0.962
Right putamen and GP	0.298	0.281	0.022	0.433
Thalamus	0.224	0.220	0.007	0.679

^1^Data presented are least squares means and *P*-values from mixed model 1-way ANOVA.

^a, b^Means lacking a common superscript letter within a row differ (*P* < 0.05).

GP, Globus Pallidus; ROI, region of interest; SEM, standard error of the mean.

#### 3.1.3 Myelin water imaging outcomes

**Figure 4:**
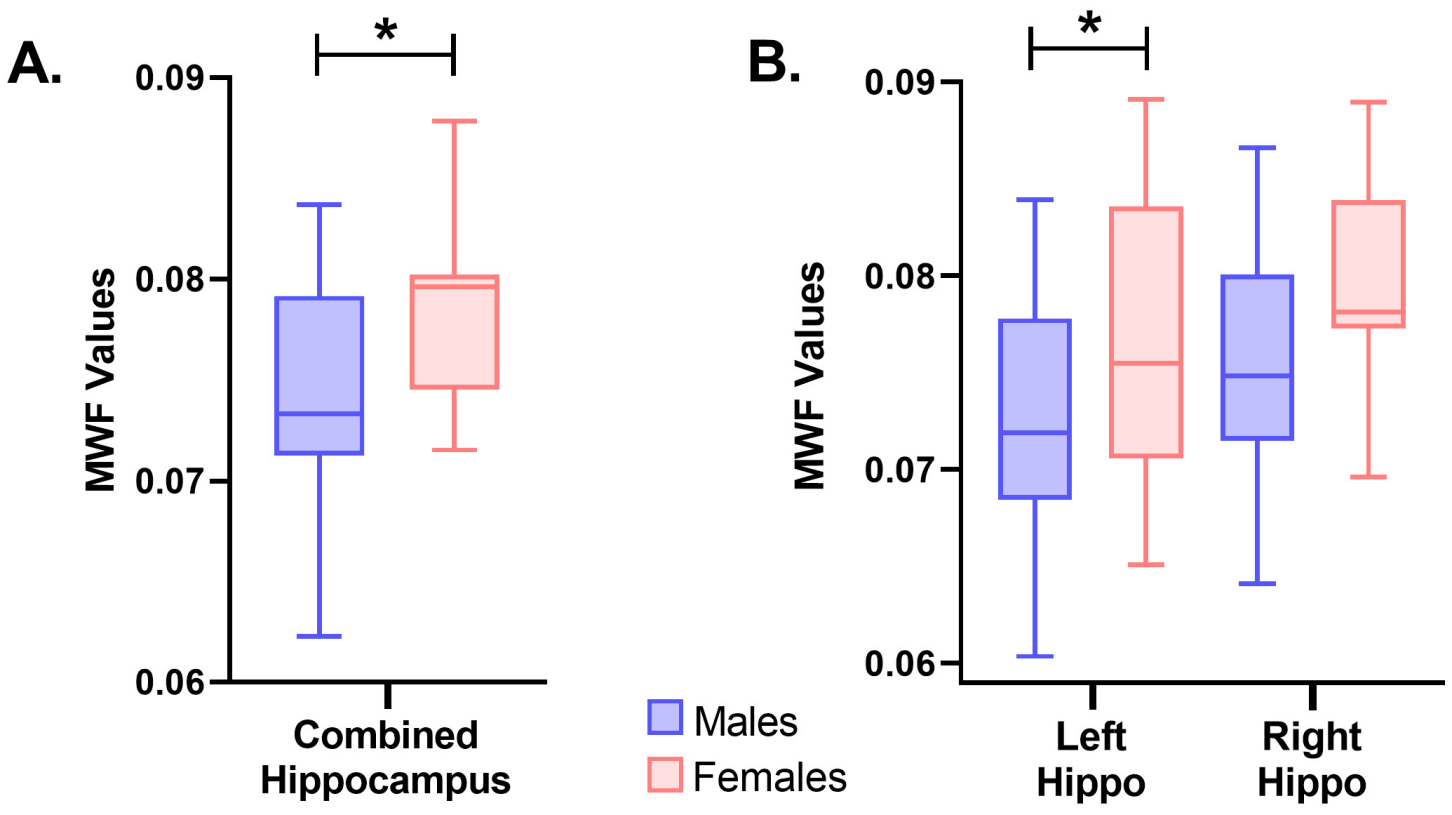
Myelin water fraction (MWF) differences between sexes in the hippocampus. **(A)** Differences between males (*n* = 11) and females (*n* = 10) were observed in MWF in the whole hippocampus (*males:0.074;females:0.079;P = 0.022*). Female pigs were observed to have higher values of MWF in the hippocampus compared to male pigs. **(B)** Additionally, differences between males (*n* = 11) and females (*n* = 10) were observed in MWF in the left hippocampus (*males:0.072;females:0.078;P = 0.038*), but not the right hippocampus (*males:0.076;females:0.080;P = 0.115*). Significance (*P* < 0.05) between means is denoted by an asterisk (*). Hippo, hippocampus.

Myelin water fraction (MWF), an established method for estimating degrees of myelination, was derived from the mcDESPOT sequence and calculated for the whole brain and specified ROIs. No differences (*P* = 0.775) were observed between male and females pigs for the whole brain and most individual ROI. Although, as pictured in Figure 4 in the whole hippocampus, a higher estimation of myelin was exhibited in female pigs as compared with male pigs (males = 0.074; females = 0.079; *P* = 0.022). This difference may provide further evidence for asymmetric variance across the hemispheres since the difference in myelination was localized to the left hippocampus (males = 0.072; females = 0.078; *P* = 0.038), and was not evident in the right hippocampus (males = 0.076; females = 0.080; *P* = 0.115). Hence, the difference observed in the whole hippocampus seems to be driven by the left side. Across all other ROI, female and male pigs exhibited no differences in MWF values (Table 3).

**Table 3:** Sex differences in myelin water fraction of four-week pigs^1^.

**ROI**	**Sex**		**Pooled SEM**	***P*-value**
	**Males/boars**	**Females/gilts**		
*n*	11	10	–	–
Whole brain	0.083	0.082	0.003	0.775
Combined caudate	0.074	0.074	0.003	0.886
Combined cortex	0.082	0.082	0.002	0.988
Combined hippocampus	0.074^a^	0.079^b^	0.003	0.022
Combined inferior colliculi	0.073	0.072	0.004	0.087
Combined internal capsules	0.119	0.112	0.008	0.442
Combined putamen and GP	0.101	0.097	0.009	0.594
Combined superior colliculi	0.073	0.079	0.003	0.120
Cerebellum	0.088	0.086	0.009	0.772
Cerebral aqueduct	0.065	0.078	0.010	0.240
Corpus callosum	0.072	0.078	0.005	0.077
Hypothalamus	0.078	0.073	0.003	0.271
Left caudate	0.073	0.078	0.007	0.530
Left cortex	0.081	0.081	0.003	0.964
Left hippocampus	0.072^a^	0.078^b^	0.005	0.038
Left inferior colliculus	0.074	0.081	0.007	0.266
Left internal capsule	0.117	0.110	0.007	0.398
Left olfactory bulb	0.055	0.052	0.011	0.545
Left putamen-globus pallidus	0.106	0.099	0.009	0.415
Left superior colliculus	0.072	0.078	0.003	0.160
Medulla	0.080	0.075	0.007	0.281
Midbrain	0.086	0.086	0.005	0.982
Nucleus accumbens	0.070	0.077	0.006	0.340
Pons	0.098	0.087	0.006	0.068
Right caudate	0.076	0.079	0.005	0.707
Right cortex	0.083	0.086	0.003	0.460
Right hippocampus	0.076	0.080	0.002	0.115
Right inferior colliculus	0.071	0.078	0.011	0.535
Right internal capsule	0.120	0.114	0.009	0.501
Right olfactory bulb	0.054	0.057	0.011	0.630
Right putamen-globus pallidus	0.096	0.095	0.010	0.815
Right superior colliculus	0.074	0.080	0.003	0.175
Substantia nigra	0.064	0.064	0.007	0.940
Thalamus	0.095	0.096	0.002	0.609

^1^Data presented are least squares means and *P*-values from mixed model 1-way ANOVA.

^a, b^Means lacking a common superscript letter within a row differ (*P* < 0.05).

GP, Globus Pallidus; ROI, region of interest; SEM, standard error of the mean.

#### 3.1.4 Histological myelin estimation of the left hippocampus

**Figure 5:**
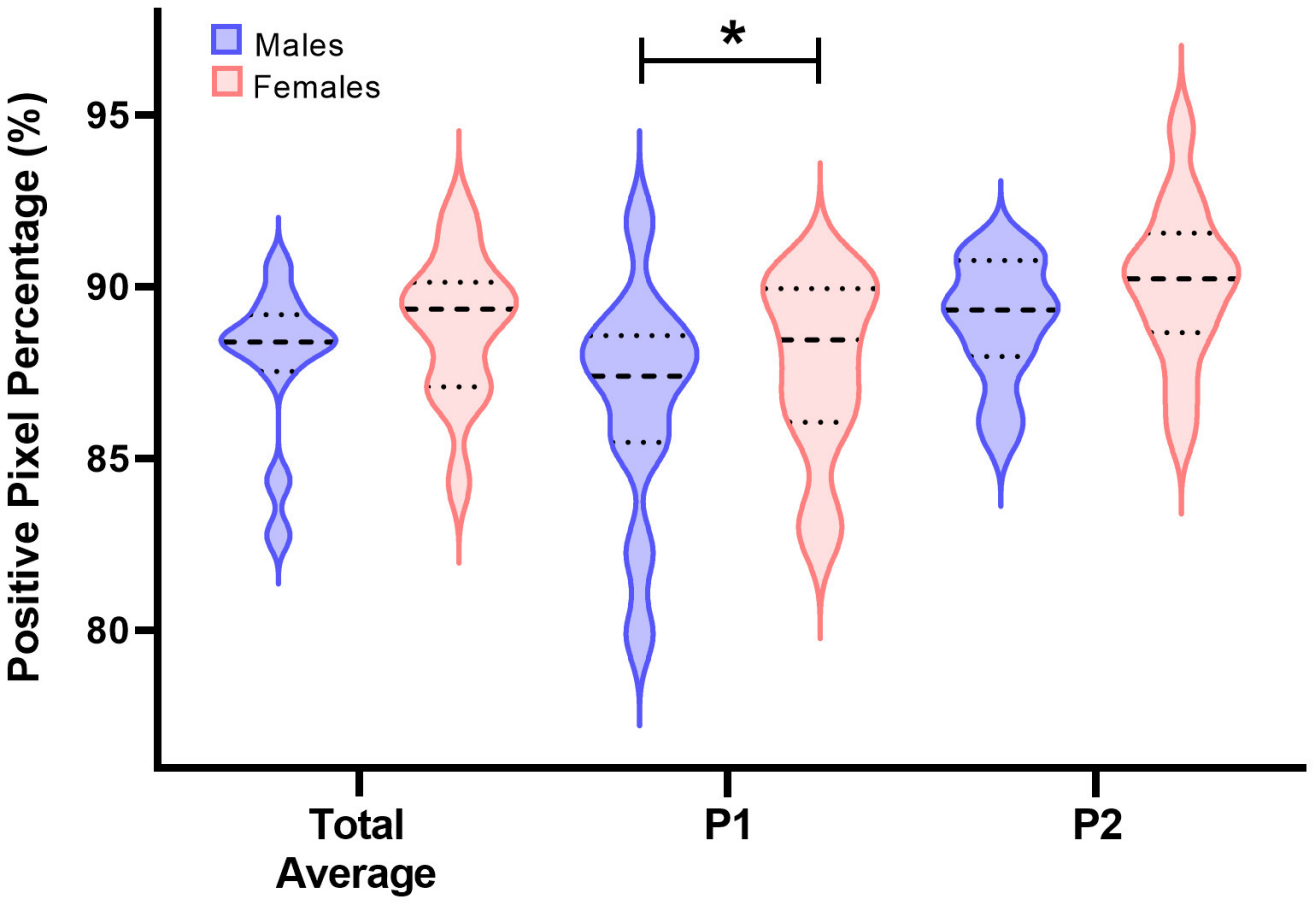
Positive pixel percentage (%) differences between sexes in the left hippocampus. Left hippocampal tissue was stained using the Gallyas silver impregnation method and myelin density was quantified using the positive pixel count percentage method. In the following violin plots, the wider the curve the higher the density of data points at that value. The bolded dashed line is the median value, the upper dashed line represents the 3rd quartile, and the lower dashed line represents the 1st quartile. On average, there were no differences between positive pixel percentage between male and female pigs. However, a difference between sexes was observed for anatomical position 1 (P1), but not anatomical position 2 (P2). In P1, female pigs were observed to have a higher positive pixel percentage in the left hippocampus compared with male pigs (*males:85.69%; females:88.53%;P = 0.024*). Significance (*P* < 0.05) between means is denoted by an asterisk (*).

Main effects of sex on myelin density within the left hippocampus as estimated using positive pixel percentage were also investigated (Figure 5, Table 4). In general, across both chosen anatomical positions, no differences were observed for hippocampal myelin density between male and female pigs (males = 87.9%; females = 88.8%; *P* = 0.333). Because two different anatomical positions were assessed, potential differences in myelin density between these positions across sexes could also be investigated. Myelin density, as captured by positive pixel percentage, was observed to be higher (*P* = 0.024) in females (88.53%) compared with males (85.69%) for position 1. For position 2, no differences were observed between sexes (males = 89.0%; females = 90.1%; *P* = 0.152).

**Table 4:** Sex differences in myelin density in the left hippocampus of young pigs as determined by positive pixel percentage^1^.

**ROI**	**Sex**		**Pooled SEM**	***P*-value**
	**Males/boars**	**Females/gilts**		
*n*	11	13	–	–
Total positive pixel count	87.90	88.84	1.032	0.333
Total negative pixel count	12.10	11.16	1.032	0.333
Average positive pixel count position 1	85.69^a^	88.53^b^	0.788	0.024
Average negative pixel count position 1	14.31^a^	11.47^b^	0.788	0.024
Average positive pixel count position 2	88.99	90.11	1.189	0.152
Average negative pixel count position 2	11.01	9.89	1.189	0.152

^1^Data presented are least squares means and *P*-values from mixed model 1-way ANOVA.

^a, b^Means lacking a common superscript letter within a row differ (*P* < 0.05).

ROI, region of interest; SEM, standard error of the mean.

### 3.2 Role of neuroimaging metrics and sex in predicting myelin density in the left hippocampus

**Figure 6:**
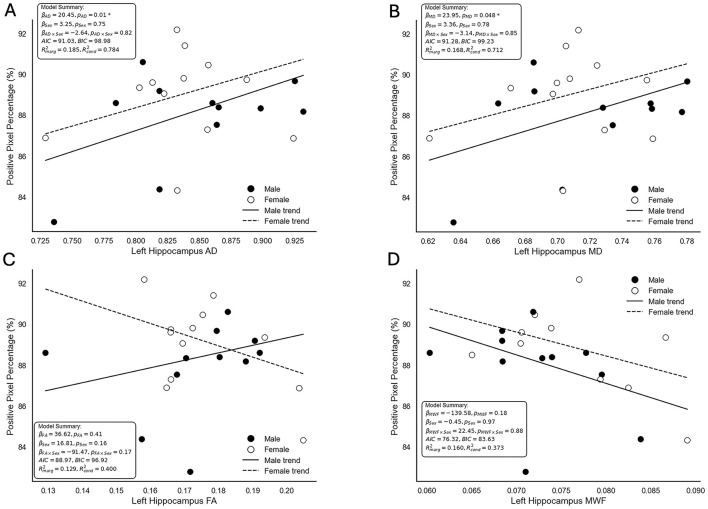
Scatterplots depicting the relationship between neuroimaging metrics and positive pixel percentage as a measure of myelin density derived from Gallyas silver histology. Displayed are the modeled relationships between positive pixel percentage and **(A)** axial diffusivity (AD), **(B)** mean diffusivity (MD), **(C)** fractional anisotropy (FA), and **(D)** myelin water fraction (MWF). Points represent individual observations, with black circles denoting male pigs and white circles denoting female pigs. Solid lines represent the trend for males, while dashed lines represent the trend for females. Model summaries, including beta coefficients (β), *p*-values (*p*), model fit indices (AIC and BIC), marginal ($R_{marg}^{2}$) and conditional ($R_{cond}^{2}$) coefficients of determination (*R*^2^), are provided for each plot. AD and MD demonstrated significant associations with positive pixel percentage, while FA and MWF did not show significant relationships. Interaction terms and sex-specific effects were not statistically significant across all models. Though for the FA **(C)**, an interaction of sex based on intersecting lines is visually observed, the low marginal and conditional *R*^2^ values denote that the current model does not explain all of the variance within the data.

The relationship between neuroimaging metrics and histologically-derived myelin density was assessed using multiple linear regression mixed-effects models. Trend lines, beta coefficients, *p*-values, AIC and BIC scores and marginal and conditional coefficients of determination (*R*^2^) for AD, MD, FA, and MWF are displayed in Figure 6. AD showed a significant positive association with positive pixel percentage, with a beta coefficient of 20.45 (*P* = 0.01). The inclusion of sex in the model did not reveal a significant main effect (β = 3.25, *P* = 0.75) or interaction term (β = −2.64, *P* = 0.82). The model had an AIC value of 91.03 and a BIC value of 98.08. MD also demonstrated a significant positive association with positive pixel percentage, with a beta coefficient of 23.95 (*P* = 0.048). Similar to the AD model, the inclusion of sex in the MD model did not yield significant main (β = 3.36, *P* = 0.78) or interactive (β = −3.14, *P* = 0.85) effects. The MD model had an AIC value of 91.28 and a BIC value of 99.23, indicating a comparable fit to the AD model. The trend lines in both AD and MD model visualizations indicate that the relationship between MRI metrics and myelin density is consistent across sexes, showing similar slopes for male and female pigs.

In contrast, FA and MWF did not show significant associations with positive pixel percentage. For FA, the beta coefficient was 36.62 (*P* = 0.41), with neither the main effect of sex (β = 16.81, *P* = 0.16) nor the interaction term (β = −91.47, *P* = 0.17) reaching significance. Similarly, MWF had a beta coefficient of −139.58 (*P* = 0.18), with no significant main effect of sex (β = −0.45, *P* = 0.97) or interaction (β = 22.45, *P* = 0.88). These results suggest no association between FA or MWF and myelin density as measured by positive pixel percentage derived from Gallyas silver histology. The FA and MWF visualizations showed more noticeable differences in trendlines between male and female pigs, but these effects were not statistically significant. The RD model did not converge using the REML method and was excluded from further interpretation and visualization in this analysis.

## 4 Discussion

Investigations into sex differences on brain development in early life have yielded inconsistent findings across species (Levine, 1966; Villablanca et al., 2000; Knickmeyer et al., 2010; Dean et al., 2018). Specifically, limited work exists on assessing these differences in domestic pigs, with studies mainly focusing on early life genetic differences (Teixeira et al., 2019; Strawn et al., 2021) and volumetric assessments (Conrad et al., 2012a,b). Given the beneficial nature of using the pig as a biomedical model for development (Lunney et al., 2021), it is imperative to establish baseline values and determine sex differences or similarities across multiple neuroimaging modalities. Additionally, there remains a question about which neuroimaging modality is most suitable and how accurately microstructural changes in the brain are depicted. Therefore, the following experimental study was designed to tackle both gaps in knowledge. By capturing multimodal neuroimaging and histological data, sex variances were captured with a specific focus on the hippocampus. To our knowledge, this is the first study to examine multiple neuroimaging modalities across sexes as well as predict which neuroimaging metric is most associated with myelin density in the domestic pig.

### 4.1 Neuroimaging and myelin density across sexes

Across neuroimaging modalities, minimal sex differences were observed, with most evidence pointing to similar structural development on both macrostructural and microstructural scales. This aligns with findings reported by Conrad et al. (2012a) who only observed differences between gilts and boars in volume at adulthood, but not at 4 weeks of age. Specifically, the 4-week time-point was chosen due to reports that this is a critical time for brain growth in the pig (Conrad et al., 2012a). Therefore, domestic pigs appear to align more closely with other species like felines and ferrets, who do not display sex differences in brain volumes during developmental periods of rapid brain growth (Villablanca et al., 2000; Garcia et al., 2024). In the human literature, evidence suggests sex differences are apparent in regional volumes across the brain with males displaying larger white matter volumes as early as infancy (Dean et al., 2018; Khan et al., 2024). However, others have found that once corrected for intracranial volume, all volume differences dissipate (Knickmeyer et al., 2014). In general, males tend to display larger absolute volumes for TBV, ICV, and across various regions at infancy (Knickmeyer et al., 2014; Dean et al., 2018; Lehtola et al., 2019; Khan et al., 2024), which was not observed in the current study for domestic pigs. Nonetheless, evidence in human literature remains mixed, and volumetric assessments alone are known to be a less sensitive method for assessing structural brain changes in development (Madan, 2021).

Other neuroimaging modalities, such as water diffusion and myelin water imaging, can provide more information on miniscule structural changes in fiber orientation and myelination. Similar to volumetric data, no differences in sex were observed across the four diffusion metrics we quantified (AD, MD, RD, and FA) in domestic pigs. This aligns with previous human studies where sex differences in FA were found in 5 year olds, but not infants (Kumpulainen et al., 2023). Other studies have reported sex differences in AD and RD across regions such as the putamen, hippocampus, and thalamus, but similar to previous findings in the domestic pig (Conrad et al., 2012a), these differences were observed at a later age (8–24 years; Kumar et al., 2012). Furthermore, in a population of infants from birth to 2 years of age, minimal sex differences were observed, all of which were isolated to fiber tracts in the right hemisphere (Geng et al., 2012). Hence, it seems that domestic pigs follow this trend of minimal diffusion differences across sex in early life, further solidifying it as a relevant biomedical model.

When assessing MWF data, there were no sex differences across any region except the hippocampus. This is of no surprise as minimal sex differences have been observed for MWF derived from mcDESPOT across the brain in a large sample of infants (Deoni et al., 2012). In the instances where they did find sex differences, such as for the genu of the corpus callosum, left frontal white matter and left temporal white matter, females were observed to have an increased developmental rate compared with males (Deoni et al., 2012). In the present study, immature female pigs (i.e., gilts) exhibited higher myelin estimated in the whole hippocampus compared to immature, intact male pigs (i.e., boars), a difference that was primarily driven by increased myelin in the left hippocampus as determined by MWF. This may reflect the observed difference in brain growth previously observed between female and male pigs (Conrad et al., 2012a). Additionally, these results may corroborate previous findings of hippocampal lateralization in rodents and humans (Jordan, 2020). In general, both left and right hippocampi contribute to spatial memory processing across species, but individual functions are believed to vary (Jordan, 2020). The left hippocampus has been associated with long-term spatial memory processing in rodents (Jordan, 2020) and recall of verbal information in humans with dementia (De Toledo-Morrell et al., 2000). On the other hand, the right hippocampus has been tied to spatial navigation across species, short-term spatial memory in mice (Jordan, 2020) and spatial recall in humans with dementia (De Toledo-Morrell et al., 2000). During a relational memory task in female human participants, hippocampal lateralization was apparent through dynamic changes in left vs. right hippocampal activation (Hopf et al., 2013). Furthermore, right lateralization has been observed in males during a spatial memory task which was associated with improved performance compared to females (Persson et al., 2013). This is contrary to our results where higher MWF values were observed in the left hippocampus of female pigs. However, Fleming and Dilger (2017) observed that between 3 and 4 weeks of age, female pigs exhibited greater exploratory behaviors compared with males, suggesting functional differences in brain development in young pigs which may be tied to the hippocampus. This was supported by Conrad et al. (2012a), who reported that female pigs attained maximum hippocampal growth by 3 weeks of age, whereas males reached their maximum at 8 weeks of age. In the present study behavioral paradigms were not implemented and only one time point was captured, making it difficult to discern how behavior and cognition may be impacted by these fluctuations in myelination, and future work is warranted to investigate these developmental differences. Therefore, in the present study further investigation into hippocampal myelination, particularly in the left side, was conducted through histological procedures and myelin density was assessed through positive pixel quantification.

Positive pixel quantification showed no sex differences in average myelin content, but the selected anatomical position may have influenced the results. Female pigs were observed to have a higher positive pixel count in position 1 compared with position 2, potentially indicating anatomical differences between sexes. Previously, sex differences were reported among the subfields of the hippocampus in humans, specifically for the parasubiculum, fimbria, hippocampal fissure, presubiculum, and hippocampal tail (Van Eijk et al., 2020). This could be the case for domestic pigs as well, although individual subfields were not quantified in the present study due to limited information on boundaries between the subfields of the pig hippocampus. Although much work has delineated hippocampal subfields using various cell staining methods (Holm and West, 1994; Saito et al., 1998; van der Beek et al., 2004; Severi et al., 2005), little to no work has distinguished these boundaries based on myelin density and across multiple anatomical positions. Future work is warranted to identify boundaries using myelin fiber density and investigate sex differences between these hippocampal subfields in the domestic pig.

### 4.2 Relationship between neuroimaging and myelin density

Across the literature, there is a resounding rhetoric that FA is closely tied to myelin content, due to similar increasing trends across childhood and into adolescence (Barnea-Goraly et al., 2005; Croteau-Chonka et al., 2016). Additionally, studies have reported an inverse relationship between FA and RD, where myelin basic protein (MBP) was positively correlated with FA and negatively correlated with RD in specific white matter structures that included the corpus callosum, fimbria, and anterior commissure (Chang et al., 2017). In other white matter tracts, no relationship with RD was observed, while a positive correlation between FA and MBP remained consistent (Chang et al., 2017). The inverse relationship between FA and RD has also been supported by studies in patients with schizophrenia (Scheel et al., 2013). This has strengthened the evidence suggesting that FA is directly tied to myelin content. However, much of this work has been criticized due to the inherent limitations of water diffusivity as a measure. Diffusion metrics are derived from water molecules within each voxel of the image, which can be influenced by a wide array of factors (Jones et al., 2013). FA, which reflects anisotropy within a voxel, can also be influenced by axon diameter and variances in fiber density (Jones et al., 2013).

Given the state of the science, it is understandable that FA did not correlate with myelin density, as reflected by positive pixel count percentage within the left hippocampus. Furthermore, this is not the first time that FA was found to be unrelated with myelin content as Arshad et al. (2016) reported similar findings in human participants. Instead, they found a correlation between MWF and RD, localized to the splenium of the corpus callosum (Arshad et al., 2016). It is important to note that other diffusion metrics (AD and MD) and histological data were not investigated (Arshad et al., 2016), which may have exhibited different results. Other studies suggest that AD and RD are more sensitive to WM tissue property changes during development (Bartzokis et al., 2012). Specifically, Bartzokis et al. (2012) observed quadratic trajectories for AD and MD in the corpus callosum across the ages of 14–93 years, reflecting typical biological fluctuations during development. However, lower FA, RD, and AD values were observed in regions with more crossing fibers, thereby highlighting their sensitivity to fiber interference (Bartzokis et al., 2012). Furthermore, most early developmental studies report that AD and MD decrease with age and exhibit a negative relationship with myelin (Kumar et al., 2012; Provenzale et al., 2012; Callow et al., 2020). This is contrary to our findings, where both AD and MD showed positive correlation with myelin density.

As backdrop to the present findings, it may be important to consider structural idiosyncrasies of the hippocampus as our selected region of interest. The hippocampus has a unique architecture and is subdivided into multiple subfields based on differences in cellular composition (Karat et al., 2023). These individual subfields contain diverse microstructural circuits, leading to variations in diffusion outcomes (Treit et al., 2018; Karat et al., 2023). For instance, Treit et al. (2018) observed varying levels of MD across the hippocampus, with most subdivisions exhibiting similar values other than the stratum lacunosum moleculare (SLM) portion of the hippocampal body, which had significantly higher MD. Similar patterns were observed for FA where the SLM and tail of the hippocampus exhibited significantly lower FA (Treit et al., 2018). Furthermore, the CA1 and CA2 regions of the hippocampus, characterized by lower neurite density, have been associated with higher values of MD (Karat et al., 2023). Due to the diverse cellular structure and circuitry across the hippocampus, diffusion metrics for the entire region may not follow expected trends. For example, when assessing a variety of brain regions, Kumar et al. (2012) observed that across most observed regions AD trended down with age. However, in the mid-hippocampus, AD increased with age, possibly a reflection of the different cytoarchitecture in this region.

When considering the whole hippocampus, MD, AD, and RD are expected to start with higher values in early life and decrease with age, with FA exhibiting an opposite trend. It is possible that at 4 weeks of age the metrics of MD and AD correlate better with myelin density. In pigs, the 4-week time-point reflects a period of rapid brain development, but the brain continues to grow in volume past 8 weeks of age into adulthood (Conrad et al., 2012a; Fil et al., 2021a). Therefore, throughout development, just as the diffusion metrics fluctuate, the parameters that most strongly correlate with myelin density may also change. Furthermore, most developmental diffusion data has focused primarily on WM tracts such as the corpus callosum, internal capsule, and longitudinal fasiculi (Schmithorst et al., 2002; Bartzokis et al., 2012; Provenzale et al., 2012), leaving the developmental trajectories of diffusion within the hippocampus less understood. The present study highlights the fluctuating trends within diffusion metrics across development and suggests that during a period of rapid brain growth, AD and MD exhibit a stronger positive correlation with histologically-derived myelin density outcomes in the hippocampus of domestic pigs. However, this positive correlation is contrary to developmental expectations, suggesting that both diffusion metrics and MWF from mcDESPOT may not accurately reflect myelination. Therefore, it is necessary to continue investigating myelination specific neuroimaging methods in order to identify a reliable and accurate method for non-invasive myelin estimation.

### 4.3 Limitations

Using non-invasive imaging methods comes with inherent limitations, as many uncertainties remain regarding what these various neuroimaging modalities truly reflect. Fluctuations in diffusion metrics across developmental stages have been associated with increasing age, maturation, and cognitive function (Schmithorst et al., 2002; Schmithorst and Yuan, 2010), but the underlying biological mechanism driving these changes remains unclear (Schmithorst and Yuan, 2010). Additionally, neuroimaging modalities specifically designed to quantify myelin content remain highly debated with conflicting results (Lee et al., 2021). Many myelin water imaging techniques, such as mcDESPOT, have been criticized for inaccuracy and lack of reproducibility, and the present study further emphasizes these limitations (Lee et al., 2021). This adds to the need for more neuroimaging studies to include molecular or histological analyses in tandem.

Although limited microstructural changes, even with histology, were observed between sexes in the present study, evidence suggests that early-life differences may be more minute and require more sensitive techniques for detection. For example, transcriptomic changes between male and female pigs have been reported as early as the 6th week of gestation (Strawn et al., 2021). Variation in gene expression and cellular signaling are also apparent between sexes at this stage of development. Additionally, similar to rats (Kühnemann et al., 1994), differences in estrogen receptor distribution have been observed across multiple brain regions with immunoreactivity more intense in female pigs (Van Leeuwen et al., 1995). Although this study was conducted after sexual maturity (Van Leeuwen et al., 1995), estrogen receptor concentrations begin to fluctuate during embryonic development in many vertebrates, including pigs, suggesting that sexual differences may begin early in life at a microscopic level (Bazer and Johnson, 2014; Bondesson et al., 2015).

Microstructural changes in the present study were captured using a classic myelin staining approach, but the method for quantification also has limitations. Positive pixel count is a widely used method (Steenstrup et al., 2000), but it has known inaccuracies. Specifically, it is known to underestimate counts compared to trained scorers (Smits et al., 2023), which may have impacted the final absolute counts in the present study. Selection of the threshold also introduced bias into results since thresholds were chosen based on visual inspection. Although, the individuals completing positive pixel quantification were trained to ensure consistency, results were still relatively subjective. Additionally, a relatively small sample size was used for quantifying myelin due to study constraints and only four representative slices were stained per subject. This small sample size as well as the shrinkage, distortion, and tears of the hippocampal tissue after preservation and staining prevented co-registration of neuroimaging modalities to histology. These challenges can all influence the capabilities of registering across imaging modalities and have been discussed in detail previously (Alyami et al., 2022). Future studies should consider including more myelin-specific neuroimaging modalities, introduce a more accurate myelin quantification method, and include a more robust representation of the brain with histology. By investigating additional brain regions, associations between myelin density and neuroimaging modalities could be further explored.

## 5 Conclusion

In conclusion, across multiple neuroimaging modalities, minimal sex differences were observed in domestic pigs at 4 weeks of age. This suggests that in studies assessing brain development at this early developmental stage, sex is not likely a confounding factor. As a result, developmental trends and findings observed in one sex are likely applicable to the other. The main sex difference observed was myelin estimation in the hippocampus, where female pigs had higher values than male pigs. This was also supported by histologically-derived myelin density, although the difference was limited to one anatomical position within the hippocampus. This may indicate the potential for sex differences across subfields of the hippocampus. However, regression analyses point to conflicting results, as MWF and FA were not correlated with myelin content as quantified by histology. Instead, diffusion metrics of axial and mean diffusivity were positively correlated with histological myelin density. These findings suggest that developmental fluctuations may influence results and current neuroimaging modalities may not accurately capture these changes. Future work should investigate sex differences in hippocampal subfields and additional myelin specific neuroimaging modalities in the domestic pig.

## Data Availability

The raw data supporting the conclusions of this article will be made available by the authors, without undue reservation.
