# Supplementary material for: Axial and mean diffusivity predict myelin density in the hippocampus of pigs during early brain development, independent of sex
Source: Front Neurosci. 2025 May 19;19:1576274. doi: 10.3389/fnins.2025.1576274 (PMC12127305; doi:10.3389/fnins.2025.1576274)
Supplement: Supplementary file 1 [file Table_1.docx]

| **Supplemental Table 1.** Absolute volume (mm^3^) of brain regions in 4-week-old pigs ^1^ | | | | | |
| --- | --- | --- | --- | --- | --- |
|  | **Sex** | |  |  | |
| **ROI** | **Males/Boars** | **Females/Gilts** | **Pooled SEM** | | ***P*-value** |
| *n* | 11 | 13 | - | | - |
| Intracranial volume | 58,612 | 58,951 | 1,746.32 | | 0.887 |
| Total brain volume | 51,476 | 51,538 | 1,651.03 | | 0.978 |
| Gray matter | 30,773 | 30,736 | 791.10 | | 0.973 |
| White matter | 20,663 | 20,608 | 897.21 | | 0.906 |
| Cerebral spinal fluid | 7,077.7 | 7,355.7 | 151.12 | | 0.206 |
| Combined caudate | 770.6 | 764.7 | 23.43 | | 0.856 |
| Combined cortex | 36,986 | 37,172 | 1,194.57 | | 0.910 |
| Combined hippocampus | 1,033.0 | 1,017.2 | 37.80 | | 0.762 |
| Combined inferior colliculi | 255.8 | 252.3 | 9.38 | | 0.787 |
| Combined internal capsules | 1,272.4 | 1,307.3 | 34.16 | | 0.471 |
| Combined putamen-globus pallidus | 401.2 | 414.5 | 10.13 | | 0.370 |
| Combined superior colliculi | 433.7 | 456.9 | 12.15 | | 0.191 |
| Cerebellum | 6,959.5 | 7,159.0 | 175.2 | | 0.428 |
| Cerebral aqueduct | 49.2 | 51.3 | 1.61 | | 0.379 |
| Corpus callosum | 500.0 | 479.6 | 18.74 | | 0.438 |
| Fourth ventricle | 110.1 | 111.7 | 4.16 | | 0.761 |
| Hypothalamus | 135.8 | 141.5 | 5.28 | | 0.338 |
| Lateral ventricle | 633.7 | 610.8 | 24.74 | | 0.514 |
| Left caudate | 396.6 | 392.3 | 13.16 | | 0.816 |
| Left cortex | 18,808 | 18,937 | 619.24 | | 0.879 |
| Left hippocampus | 520.6 | 509.9 | 17.86 | | 0.665 |
| Left inferior colliculus | 127.2 | 125.9 | 5.25 | | 0.856 |
| Left internal capsule | 679.1 | 694.6 | 16.80 | | 0.524 |
| Left olfactory bulb | 1,123.8 | 1,113.6 | 47.90 | | 0.878 |
| Left putamen-globus pallidus | 205.3 | 213.3 | 5.84 | | 0.353 |
| Left superior colliculus | 213.4 | 222.6 | 5.65 | | 0.261 |
| Medulla | 1,940.0 | 1,939.1 | 58.44 | | 0.991 |
| Midbrain | 2,373.2 | 2,383.2 | 65.65 | | 0.912 |
| Nucleus accumbens | 51.5 | 52.7 | 2.19 | | 0.578 |
| Pons | 1,560.2 | 1,611.1 | 36.72 | | 0.344 |
| Right caudate | 373.1 | 372.3 | 10.28 | | 0.957 |
| Right cortex | 18,412 | 18,461 | 587.79 | | 0.951 |
| Right hippocampus | 511.6 | 507.3 | 20.17 | | 0.876 |
| Right inferior colliculus | 128.4 | 126.4 | 4.51 | | 0.747 |
| Right internal capsule | 590.4 | 612.6 | 17.67 | | 0.374 |
| Right olfactory bulb | 1,149.3 | 1,141.3 | 48.79 | | 0.905 |
| Right putamen-globus pallidus | 195.4 | 200.9 | 4.85 | | 0.437 |
| Right superior colliculus | 218.9 | 232.9 | 7.16 | | 0.178 |
| Substantia nigra | 42.3 | 43.4 | 1.36 | | 0.559 |
| Thalamus | 1,543.0 | 1,561.6 | 35.20 | | 0.708 |
| ^1^Data presented are least squares means and *P*-values from mixed model 1-way ANOVA. Abbreviations: ROI, region of interest; SEM, standard error of the mean. | | | | | |

| **Supplemental Table 2.** Axial diffusivity (AD; × 10⁻³ /mm²/s) of brain regions in 4-week-old pigs^1^ | | | | |
| --- | --- | --- | --- | --- |
|  | **Sex** | |  |  |
| **ROI** | **Males/Boars** | **Females/Gilts** | **Pooled SEM** | ***P*-value** |
| *n* | 11 | 12 | - | - |
| Corpus callosum | 0.918 | 0.914 | 0.035 | 0.925 |
| Hippocampus | 0.848 | 0.834 | 0.014 | 0.457 |
| Internal capsules | 0.828 | 0.839 | 0.011 | 0.518 |
| Left caudate | 0.854 | 0.837 | 0.015 | 0.343 |
| Left cortex | 0.830 | 0.807 | 0.013 | 0.228 |
| Left hippocampus | 0.846 | 0.836 | 0.016 | 0.667 |
| Left internal capsule | 0.834 | 0.831 | 0.019 | 0.913 |
| Left putamen-globus pallidus | 0.803 | 0.793 | 0.011 | 0.532 |
| Right caudate | 0.835 | 0.814 | 0.011 | 0.168 |
| Right cortex | 0.842 | 0.824 | 0.012 | 0.134 |
| Right hippocampus | 0.849 | 0.843 | 0.009 | 0.642 |
| Right internal capsule | 0.823 | 0.837 | 0.017 | 0.464 |
| Right putamen-globus pallidus | 0.813 | 0.802 | 0.011 | 0.494 |
| Thalamus | 0.756 | 0.745 | 0.012 | 0.545 |
| ^1^Data presented are least squares means and *P*-values from mixed model 1-way ANOVA. Abbreviations: ROI, region of interest; SEM, standard error of the mean. | | | | |

| **Supplemental Table 3.** Mean diffusivity (MD; × 10⁻³ /mm²/s) of brain regions in 4-week-old pigs^1^ | | | | |
| --- | --- | --- | --- | --- |
|  | **Sex** | |  |  |
| **ROI** | **Males/Boars** | **Females/Gilts** | **Pooled SEM** | ***P*-value** |
| *n* | 11 | 12 | - | - |
| Corpus callosum | 0.742 | 0.738 | 0.026 | 0.911 |
| Hippocampus | 0.722 | 0.706 | 0.014 | 0.299 |
| Internal capsules | 0.550 | 0.552 | 0.008 | 0.873 |
| Left caudate | 0.717 | 0.703 | 0.015 | 0.296 |
| Left cortex | 0.734 | 0.712 | 0.012 | 0.216 |
| Left hippocampus | 0.720 | 0.707 | 0.015 | 0.491 |
| Left internal capsule | 0.558 | 0.549 | 0.009 | 0.449 |
| Left putamen-globus pallidus | 0.628 | 0.611 | 0.006 | 0.079 |
| Right caudate | 0.706 | 0.683 | 0.009 | 0.060 |
| Right cortex | 0.742 | 0.726 | 0.012 | 0.159 |
| Right hippocampus | 0.725 | 0.705 | 0.013 | 0.168 |
| Right internal capsule | 0.542 | 0.541 | 0.011 | 0.979 |
| Right putamen-globus pallidus | 0.609 | 0.616 | 0.007 | 0.473 |
| Thalamus | 0.612 | 0.613 | 0.006 | 0.889 |
| ^1^Data presented are least squares means and *P*-values from mixed model 1-way ANOVA. Abbreviations: ROI, region of interest; SEM, standard error of the mean. | | | | |

| **Supplemental Table 4.** Radial diffusivity (RD; × 10⁻³ /mm²/s) of brain regions in 4-week-old pigs^1^ | | | | |
| --- | --- | --- | --- | --- |
|  | **Sex** | |  |  |
| **ROI** | **Males/Boars** | **Females/Gilts** | **Pooled SEM** | ***P*-value** |
| *n* | 11 | 12 | - | - |
| Corpus callosum | 0.654 | 0.650 | 0.023 | 0.897 |
| Hippocampus | 0.659 | 0.642 | 0.014 | 0.236 |
| Internal capsules | 0.403 | 0.405 | 0.008 | 0.850 |
| Left caudate | 0.648 | 0.635 | 0.014 | 0.309 |
| Left cortex | 0.686 | 0.665 | 0.011 | 0.213 |
| Left hippocampus | 0.656 | 0.643 | 0.015 | 0.393 |
| Left internal capsule | 0.411 | 0.408 | 0.008 | 0.749 |
| Left putamen-globus pallidus | 0.541 | 0.520 | 0.007 | 0.066 |
| Right caudate | 0.640 | 0.618 | 0.009 | 0.059 |
| Right cortex | 0.692 | 0.677 | 0.013 | 0.177 |
| Right hippocampus | 0.663 | 0.642 | 0.013 | 0.137 |
| Right internal capsule | 0.400 | 0.401 | 0.012 | 0.957 |
| Right putamen-globus pallidus | 0.507 | 0.522 | 0.013 | 0.310 |
| Thalamus | 0.539 | 0.537 | 0.008 | 0.866 |
| ^1^Data presented are least squares means and *P*-values from mixed model 1-way ANOVA. Abbreviations: ROI, region of interest; SEM, standard error of the mean. | | | | |
